# Association of nutritional status indices with clinical outcomes in acute pancreatitis

**DOI:** 10.3389/fnut.2025.1662958

**Published:** 2025-09-08

**Authors:** Meltem Genc, Eda Nur Duran, Iskender Ekinci, Mehmet Bayram, Hafize Uzun, Omur Tabak

**Affiliations:** ^1^Department of Internal Medicine, Kanuni Sultan Süleyman Training and Research Hospital, Health Sciences University, Istanbul, Türkiye; ^2^Department of Internal Medicine, Faculty of Medicine, Bezmialem Vakif University, Istanbul, Türkiye; ^3^Department of Medical Biochemistry, Faculty of Medicine, Istanbul Atlas University, Istanbul, Türkiye

**Keywords:** acute pancreatitis, clinical outcomes, Controlling Nutritional Status score, nutritional management, Prognostic Nutritional Index

## Abstract

**Background/Objectives:**

Acute pancreatitis (AP) is an inflammatory condition marked by pancreatic tissue damage due to the activation of digestive enzymes in the pancreas, triggered by various factors. Nutritional status is considered an essential factor in the management and progression of AP. The Prognostic Nutritional Index (PNI) and Controlling Nutritional Status (CONUT) score are used to assess nutritional status and may have prognostic value in AP. This study aimed to investigate whether PNI and CONUT scores at admission are associated with clinical outcomes in patients with AP.

**Methods:**

A retrospective analysis was conducted on 261 acute pancreatitis patients admitted between 2016 and 2021. Statistical analyses included normality assessment (Shapiro–Wilk), group comparisons (Student’s *t*-test, Mann–Whitney U, chi-square/Fisher’s exact test), correlation analyses (Pearson/Spearman), logistic regression for predictors of complications, and ROC curve analysis for cut-off determination.

**Results:**

Patients were categorized into two groups: those with (*n* = 132) and those without complications (*n* = 129). No significant difference was observed in PNI scores between the two groups (*p* > 0.05). However, CONUT scores were significantly higher in patients with complications (*p* = 0.012). In cases with necrosis, PNI scores were substantially lower (*p* = 0.036), whereas CONUT scores were significantly higher (*p* = 0.006). A significant inverse correlation was found between PNI and CONUT scores (*r* = −0.584, *p* < 0.001). The optimal CONUT cut-off value for predicting complications was ≥1.5, with a sensitivity of 67.4% and a specificity of 47.3%.

**Conclusion:**

This study demonstrates that while PNI scores did not significantly differ between AP patients with and without complications, lower PNI and higher CONUT scores were significantly associated with the presence of pancreatic necrosis. Moreover, the CONUT score was significantly higher in patients who developed complications, suggesting its potential utility as a prognostic tool. These findings highlight the importance of early assessment of nutritional status in the clinical management of AP. The CONUT score, in particular, may help identify patients at risk for worse outcomes and guide timely nutritional interventions to improve prognosis.

## 1 Introduction

Acute pancreatitis (AP) is a sudden inflammatory disorder of the pancreas triggered by a range of etiological factors, leading to complex inflammatory pathways that result in varying clinical presentations and impact patient outcomes (1). Although gallstones and alcohol are most commonly involved in the etiology of AP, many etiological factors such as hypertriglyceridemia, hypercalcemia, some drugs (incretin mimetics, etc.), and genetic and autoimmune factors are also involved (2). According to the revised Atlanta classification, AP is categorized as mild (no organ failure and no local or systemic complications), moderately severe (transient organ failure or local/systemic complications without persistent organ failure), or severe (persistent organ failure lasting more than 48 h) (13). While mild cases are associated with very low mortality, moderately severe and severe forms account for approximately 15%–25% of cases, with severe cases showing mortality rates as high as 36%–50% (4). These classifications are important for prognosis and treatment management.

The disease is typically characterized by abdominal pain, elevated pancreatic enzymes, increased inflammatory mediators, and edematous or necrotizing pancreatic tissue on radiological imaging (5). The diagnosis of the disease is made in the presence of at least two of the following three criteria: typical sudden onset of abdominal pain, pancreatic enzymes above three times the upper limit, and inflammatory changes on pancreatic imaging (5).

Necrotizing pancreatitis is a more severe form with areas of parenchymal or peripancreatic necrosis and a more prominent systemic inflammatory response. Along with increased inflammation, hypermetabolic response, and protein catabolism, the need for energy and nutrients increases significantly in patients. Therefore, nutritional management, especially in the necrotizing form, is one of the fundamental approaches that directly impact the clinical course (6, 7). Since malnutrition has adverse effects on prognosis, it is crucial to evaluate nutritional status and provide appropriate support to these patients. The prognostic nutritional index (PNI) and nutritional status control (CONUT) scores, which serve as nutritional markers, have been utilized in numerous studies to examine the disease course.

While numerous studies have evaluated these markers in malignancies (3, 8), their prognostic significance in AP has not been thoroughly studied. Although nutritional status is known to affect AP outcomes, studies evaluating prognostic indices like PNI and CONUT in this context are limited. Most existing research focuses on single markers or other diseases, with few addressing their role in predicting complications such as pancreatic necrosis.

l This study aimed to investigate whether nutritional status at the time of hospital admission, as assessed by PNI and CONUT scores, is associated with the development of complications, inflammatory burden, and clinical severity in patients diagnosed with AP. Our study aims to fill this gap by assessing the association between these nutritional scores at admission and clinical outcomes in a larger patient cohort.

## 2 Materials and methods

The Ethics Committee approval from Istanbul Kanuni Sultan Suleyman Training and Research Hospital, dated December 9, 2021, and decision number 2021.12.288, was received for this study. This study was conducted by the principles outlined in the Declaration of Helsinki.

### 2.1 Study design and population

In this retrospective study, all patients aged 18–65 years who were diagnosed at Istanbul Kanuni Sultan Suleyman Training and Research Hospital between 2016 and 2021 were evaluated. A total of 261 patients were diagnosed with AP and were included in the study.

### 2.2 Inclusion criteria

Patients aged between 18 and 65 years who were diagnosed with AP according to the revised Atlanta classification were included in the study. The diagnosis of AP was based on the presence of at least two of the following criteria: (i) characteristic abdominal pain, (ii) serum amylase and/or lipase levels at least three times the upper limit of normal, and (iii) imaging findings consistent with AP (3).

### 2.3 Exclusion criteria

Malignancy, leukemia, triglyceride levels above 500 mg/dL, protein-losing enteropathies, cachectic patients, and patients with a history of cerebrovascular events were excluded. Additionally, patients with nephrotic proteinuria (e.g., chronic renal failure, diabetic or hypertensive nephropathy) were excluded. Age, gender, duration of hospitalization, comorbidities, hemogram, and biochemistry values were evaluated. Radiological findings (e.g., edema) and local complications (pseudocyst, abscess, necrosis) were assessed according to the revised Atlanta classification. Additional clinical findings, such as pleural or pericardial effusion, Gray Turner sign, ileus, and atelectasis, were documented; however, these were not evaluated as systemic complications according to the revised Atlanta classification, which defines systemic complications based on persistent organ dysfunction. However, organ dysfunction scoring using the Modified Marshall system, which is recommended in the revised Atlanta classification, could not be applied due to the retrospective design and insufficient availability of physiologic parameters.

Prognostic nutritional index and CONUT scores were calculated using the patients’ data at the time of admission, and the relationship between these scores and the development of complications was examined. Nutritional status was evaluated using two widely accepted indices: the PNI and the CONUT score. The PNI was calculated using the formula: (10 × serum albumin [g/dL]) + (0.005 × total lymphocyte count [/mm^3^]).

**Table:** 

PNI Value	Nutritional Status
PNI ≥ 50	Normal
50 > PNI ≥ 45	Mild malnutrition
45 > PNI ≥ 40	Moderate malnutrition
PNI < 40	Severe malnutrition

CONUT score is calculated as albumin score + lymphocyte score + total cholesterol score.

**Table:** 

	Normal	Mild	Moderate	Severe
Albumin (g/dL)	≥3.5 (0 points)	3.0–3.4 (2 points)	2.5–2.9 (4 points)	<2.5 (6 points)
Total lymphocyte (mm^3^)	≥1600 (0 points)	1200–1599 (1 point)	800–1199 (2 points)	<800 (3 points)
Total cholesterol (mg/dL)	≥180 (0 points)	140–179 (1 point)	100–139 (2 points)	<100 (3 points)
Total score	**0–1**	**2–4**	**5–8**	**9–12**

Prognostic nutritional index and CONUT scores were calculated using the patients’ data at the time of admission, and the relationship between these scores and the development of complications was examined. All parameters used for the calculation of PNI and CONUT, including serum albumin, total cholesterol, and lymphocyte count, were obtained from fasting venous blood samples at the time of hospital admission. Serum samples were collected in serum separator tubes, and complete blood counts were performed on EDTA-anticoagulated whole blood.

### 2.4 Clinical evaluation

Magnetic resonance cholangiopancreatography (MRCP) was performed in patients who could not be differentiated as having biliary or non-biliary disease. According to USG, CT, and MRCP findings, those with gallbladder stones, sludge, or dilatation of the choledochal duct were considered biliary, and the others were deemed non-biliary. According to imaging reports, *edema* was seen in 98 patients, necrosis in 8 patients, pseudocyst in 13 patients, and combinations of these lesions in some patients. Pleural effusion in 12 patients, pericardial effusion in 1 patient, *gray* turner in 1 patient, atelectasis in one of the patients with pleural effusion, and ileus in one of the patients with pleural effusion, and this patient was referred to the intensive care unit. Whether the effusion was due to pancreatitis was evaluated by examining the previous chest radiographs or thorax CT scans of the patients. The type of discharge was determined according to outpatient clinic control, intensive care referral, and exitus.

Early fluid resuscitation and enteral nutritional support were not systematically implemented during the initial hospitalization period and were therefore excluded from the analysis.

### 2.5 Laboratory parameters

All blood samples were collected from the antecubital vein between 08:00 and 11:30 a.m. following an 8–12 h fasting period. Biochemical parameters including amylase, lipase, aspartate aminotransferase (AST), alanine aminotransferase (ALT), alkaline phosphatase (ALP), gamma-glutamyl transferase (GGT), total and direct bilirubin, lactate dehydrogenase (LDH), c-reactive protein (CRP), glucose, urea, creatinine, calcium, albumin, total cholesterol, low-density lipoprotein (LDL), triglycerides, thyroid stimulating hormone (TSH), T4, vitamin B12, and 25-OH vitamin D were obtained using gel-separator biochemistry tubes (Vacusera, Istanbul, Turkey). After clot formation, serum samples were separated by centrifugation at 4000 × *g* for 10 min and analyzed within 4 h using the Roche Modular Analytics Cobas 8000 Immunoassay Analyzer (Roche Diagnostics GmbH, Mannheim, Germany). Complete blood counts were performed on whole blood samples collected in EDTA tubes (Vacusera, Istanbul, Turkey) using the Focusing Flow-DC method on a Mindray BC-6200 automated hematology analyzer (Shenzhen, China).

### 2.6 Statistical analysis

Statistical analyses were performed using IBM SPSS Statistics version 22 (IBM Corp., Armonk, NY, USA). A *post hoc* power analysis was conducted using G*Power 3.1 software to determine whether the sample size was sufficient to detect significant differences in the primary outcomes. The analysis indicated that the study had adequate power (≥80%) for the main comparisons. During the evaluation of the study data, the suitability of the parameters for a normal distribution was assessed using the Shapiro-Wilk test. In addition to descriptive statistical methods (mean, standard deviation, frequency), the Student *t*-test was used for comparisons of parameters with normal distribution between two groups. For variables that did not meet the assumption of normality, median and interquartile ranges (IQR) were reported, and non-parametric tests (Mann–Whitney U test) were used for comparisons of parameters without normal distribution between two groups. The chi-square test and Fisher Freeman Halton test were used to compare qualitative data. Pearson’s correlation analysis was used to analyze the relationships between parameters that conformed to a normal distribution, and Spearman’s rho correlation analysis was used to analyze the relationships between parameters that did not conform to a normal distribution. Logistic regression analysis was applied for multivariate analysis. For each predictor, we report the logistic regression coefficient (β), its standard error (SE), the odds ratio [Exp(β)] with a 95% confidence interval, and the *p*-value. The ROC curve was drawn to determine the cut-off point. Significance was evaluated at *p* < 0.05 level.

## 3 Results

A total of 261 patients hospitalized with a diagnosis of AP between 2016 and 2021 were included in the study. In terms of demographics, the mean age of the participants was 54.51 ± 17.56 years, with 53.3% of the participants being female and 46.7% being male (Table 1). Accordingly, the patients were divided into two groups based on the development of complications; 129 (49.4%) had no complications, and 132 (50.6%) developed complications. As shown in Table 2, edema (74.2%) was the most common local finding in the group with complications. Among imaging findings, edema was frequently observed; however, according to the revised Atlanta classification, it is not categorized as a local complication. In addition, other pathologies, such as pseudocyst (14.4%), necrosis (13.6%), and abscess (3%), were also reported significantly in those who developed complications (Table 2).

**TABLE 1:** Comparison of demographic characteristics between patients with and without complications of acute pancreatitis (*n* = 261).

Parameter	None (*n* = 129)	Present (*n* = 132)	Total (*n* = 261)	*P*-value
Age (mean ± SD)	54.49 ± 18.26	54.53 ± 16.92	54.51 ± 17.56	0.985^a^
Gender				0.673^b^
Female	67 (51.9%)	72 (54.5%)	139 (53.3%)	
Male	62 (48.1%)	60 (45.5%)	122 (46.7%)	
Age group				0.356^b^
<65	84 (65.1%)	93 (70.5%)	177 (67.8%)	
≥65	45 (34.9%)	39 (29.5%)	84 (32.2%)	
Presence of comorbidity				0.156^b^
None	40 (31.0%)	52 (39.4%)	92 (35.2%)	
Present	89 (69.0%)	80 (60.6%)	169 (64.8%)	
Comorbidities				
DM	34 (26.4%)	26 (19.7%)	60 (23.0%)	0.201^b^
HT	45 (34.9%)	40 (30.3%)	85 (32.6%)	0.430^b^
CKD	6 (4.7%)	3 (2.3%)	9 (3.4%)	–^c^
CHF	7 (5.4%)	5 (3.8%)	12 (4.6%)	–^c^
Other	58 (45.0%)	42 (31.8%)	100 (38.3%)	*0.029^b^

DM, diabetes mellitus; HT, hypertension; CKD, chronic kidney disease; CHF, congestive heart failure. Data are presented as mean ± SD for normally distributed variables or median (IQR) for non-normally distributed variables; categorical variables as *n* (%). *P*-values are annotated with superscripts indicating the statistical test: ^a^*t*-test; ^b^χ^2^ test; ^c^Fisher’s exact test (used when expected counts < 5). Statistically significant *p*-values are marked with * (*p* < 0.05).

**TABLE 2:** Distribution of radiological and clinical complications in patients with and without complications of acute pancreatitis.

CT finding	No complication (*n* = 129)	With complication (*n* = 132)	Total (*n* = 261)	*P*
Edema	0 (0%)	98 (74.2%)	98 (37.5%)	
Pseudocyst	0 (0%)	19 (14.4%)	19 (7.3%)	
Abscess	0 (0%)	4 (3.0%)	4 (1.5%)	
Necrosis	0 (0%)	18 (13.6%)	18 (6.9%)	
**Systemic complications:**				
**Systemic complication**	**No complication (*n* = 129)**	**With complication (*n* = 132)**	**Total (*n* = 261)**	***P***
Pleural effusion	0 (0%)	10 (7.6%)	10 (3.8%)	
Pericardial effusion	0 (0%)	1 (0.8%)	1 (0.4%)	
Gray turner	0 (0%)	1 (0.8%)	1 (0.4%)	
Ileus	0 (0%)	1 (0.8%)	1 (0.4%)	
Pleural + atelectasis	0 (0%)	1 (0.8%)	1 (0.4%)	

CT, Computed Tomography. Data are presented as *n* (%). No statistical comparison was made due to small subgroup sizes. Edema is not classified as a local complication per the revised Atlanta classification [Banks et al. (3)].

When laboratory findings were analyzed according to the development of complications, the levels of amylase, leukocytes, neutrophils, and glucose in those who developed complications were found to be statistically significantly higher. In contrast, ALP and eosinophil levels were found to be substantially lower than those who did not develop complications (*p* < 0.05). ALP and eosinophil levels were significantly lower (*p* < 0.05) (Table 3).

**TABLE 3:** Comparison of laboratory parameters between patients with and without complications in acute pancreatitis (*n* = 261).

Parameter	No complication (*n* = 129) Mean ± SD (median)	With complication (*n* = 132) Mean ± SD (median)	*P*
Amylase (U/L)	866.51 ± 926.64 (619)	1349.58 ± 1334.11 (946)	*0.020
Lipase (U/L)	1811.82 ± 2133.75 (1143)	2257.22 ± 2685.76 (1376)	0.432
AST (U/L)	101.74 ± 159.12 (28)	106.05 ± 202.21 (36.5)	0.455
ALT (U/L)	94.66 ± 141.68 (28)	93.48 ± 140.1 (31)	0.675
GGT (U/L)	131.69 ± 180.21 (50)	123.24 ± 153.87 (51.5)	0.907
ALP (U/L)	116.44 ± 61.83 (94)	103.61 ± 54.05 (88.5)	*0.042
Total bilirubin (mg/dL)	1.07 ± 1.35 (0.5)	0.93 ± 0.8 (0.6)	0.263
Direct bilirubin (mg/dL)	0.67 ± 1.12 (0.2)	0.44 ± 0.52 (0.2)	0.978
CRP (mg/dL)	32.38 ± 51.96 (10.3)	46.08 ± 73.28 (10.5)	0.466
Glucose (mg/dL)	139.12 ± 70.2 (118)	150.73 ± 58.9 (142)	*p < 0.001
Leukocyte (×10^3^/μL)	10722.4 ± 3516.56 (10540)	13045.68 ± 4607.41 (12320)	*p < 0.001
Neutrophil (×10^3^/μL)	7658.53 ± 3071.95	10230.34 ± 4246.54	*p < 0.001
Lymphocyte (×10^3^/μL)	1995.35 ± 993.33	1793.18 ± 1046.49	0.111
Basophil (×10^3^/μL)	41.63 ± 45.99 (30)	38.41 ± 25.17 (30)	0.875
Monocyte (×10^3^/μL)	772.48 ± 869.84 (660)	800.34 ± 545.15 (715)	0.183
Eosinophil (×10^3^/μL)	158.84 ± 160.92 (110)	84.32 ± 113.59 (40)	*p < 0.001
Albumin (g/dL)	4.07 ± 0.49	4.07 ± 0.5	0.911
Calcium (mg/dL)	9.2 ± 0.59 (9.2)	9.18 ± 0.63 (9.2)	0.812
Cholesterol (mg/dL)	160.87 ± 32.9	156 ± 39.46	0.281
LDL (mg/dL)	97.8 ± 28.82	95.2 ± 34.28	0.508
Triglyceride (mg/dL)	118.57 ± 47.48	108.92 ± 47.82	0.103
Hemoglobin (g/dL)	13.35 ± 1.88	13.81 ± 2.02	0.059
Hematocrit (%)	39.78 ± 5.25	41.04 ± 5.49	0.059
Platelet (×10^3^/μL)	246875.97 ± 66735.25	264234.85 ± 78447.32	0.055
Urea (mg/dL)	36.11 ± 18.54 (31)	34.74 ± 19.18 (30)	0.465
Creatinine (mg/dL)	0.98 ± 0.74 (0.8)	0.92 ± 0.82 (0.8)	0.679
Procalcitonin (ng/mL)	1.54 ± 8.49 (0.1)	1.25 ± 4.72 (0.1)	0.057

All *p*-values in this table were calculated using the non-parametric Mann–Whitney U test.

*Statistically significant difference (*p* < 0.05). AST, aspartate aminotransferase; ALT, alanine aminotransferase; GGT, gamma-glutamyl transferase; ALP, alkaline phosphatase; CRP, C-reactive protein; LDL, low-density lipoprotein.

The median hospital stay was significantly more extended in patients who developed complications (median 7 days) compared to those without complications (median 5 days) (*p* < 0.001). While there was no significant difference in PNI scores between the two groups (*p* = 0.338), CONUT scores were significantly higher in patients with complications (*p* = 0.012). The rate of moderate to severe malnutrition, based on the CONUT classification, was also higher in the complication group, whereas PNI-based classifications did not show a significant difference.

Prognostic nutritional index was significantly lower in women compared to men (*p* = 0.001), and the prevalence of severe malnutrition was higher in females (10.1%) than in males (5.7%). However, CONUT scores did not differ significantly between the sexes. Advantages were associated with lower PNI and higher CONUT scores (*p* < 0.001 for both), indicating an increased nutritional vulnerability in older patients. Similarly, patients with biliary etiology exhibited lower PNI and higher CONUT scores compared to those with non-biliary causes (*p* < 0.001), and the rates of severe and moderate malnutrition were significantly higher in this group.

Patients with pancreatic necrosis also showed significantly lower PNI scores (*p* = 0.036) and higher CONUT scores (*p* = 0.006), suggesting a potential link between impaired nutritional status and the development of local complications in AP.

**FIGURE 1:**
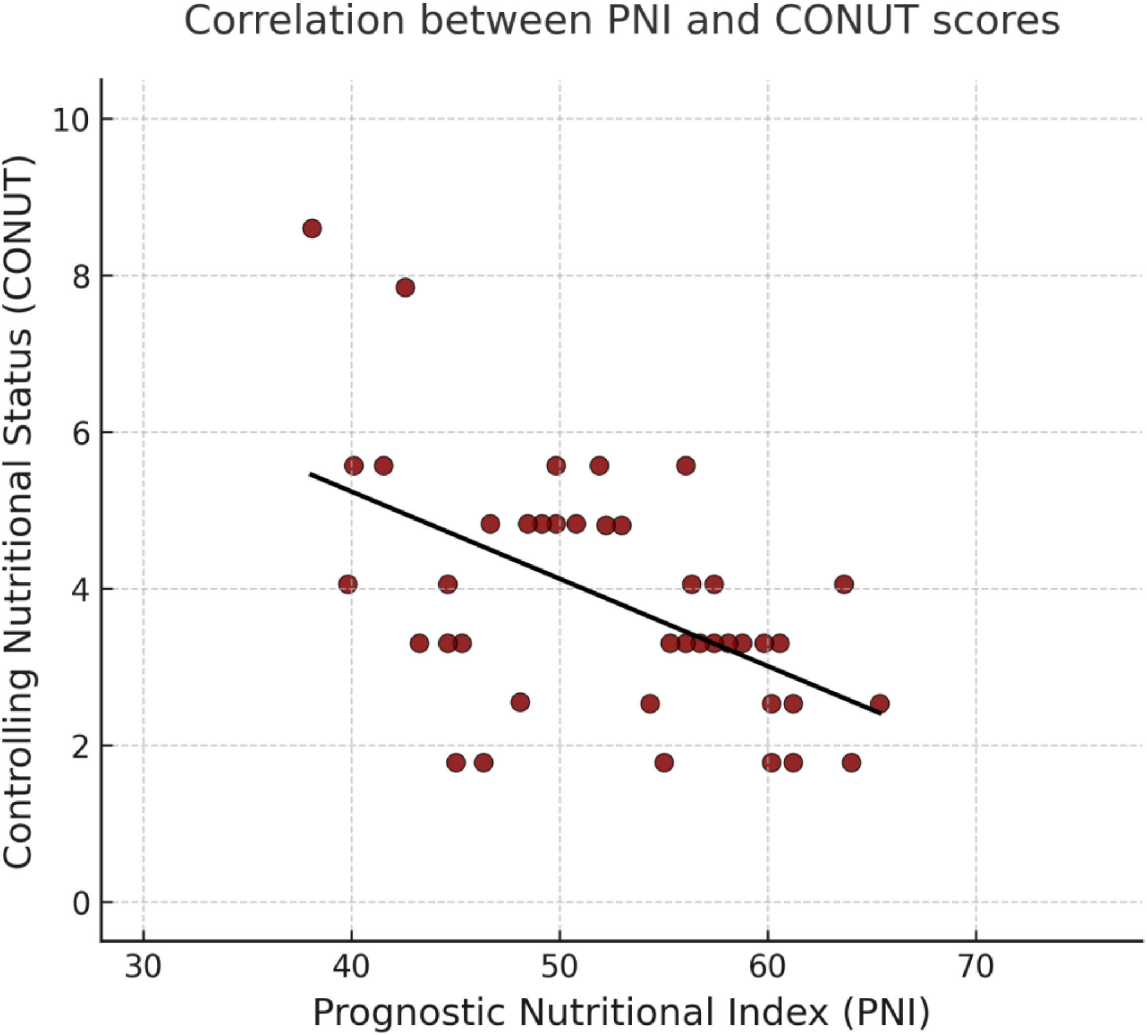
Correlation between PNI and CONUT mean scores.

Correlation analyses between nutritional indices (CONUT and PNI) and laboratory parameters are presented in Table 4. Correlation analysis revealed that PNI was inversely associated with age, AST, ALT, GGT, ALP, bilirubin, CRP, neutrophils, and urea and positively associated with albumin, lymphocytes, monocytes, basophils, eosinophils, hemoglobin, platelets, and hematocrit (all *p* < 0.05). A strong inverse correlation was also found between PNI and CONUT scores (*r* = −0.584, *p* < 0.001). Additionally, CONUT scores were positively correlated with amylase, liver enzymes, bilirubin, neutrophils, and procalcitonin and negatively correlated with albumin, total cholesterol, LDL, lymphocytes, monocytes, basophils, eosinophils, hemoglobin, platelets, and hematocrit (*p* < 0.05). The correlation between PNI and CONUT mean scores is presented in Figure 1.

**TABLE 4:** Correlation between nutritional indices (CONUT and PNI) and laboratory parameters in patients with acute pancreatitis.

Parameter	Correlation (ρ)	*P*
Age	0.117	0.059
Amylase	0.173	*0.005
Lipase	0.112	0.071
AST	0.194	*0.002
ALT	0.151	*0.014
GGT	0.152	*0.014
ALP	0.136	*0.028
Total bilirubin	0.268	*p < 0.001
Direct bilirubin	0.298	*p < 0.001
Calcium	−0.084	0.174
Albumin	−0.340	*p < 0.001
LDL	−0.497	*p < 0.001
Cholesterol	−0.519	*p < 0.001
Triglyceride	−0.120	0.053
CRP	0.020	0.748
Leukocyte	−0.051	0.416
Neutrophil	0.189	*0.002
Lymphocyte	−0.599	*p < 0.001
Monocyte	−0.180	*0.004
Basophil	−0.352	*p < 0.001
Eosinophil	−0.478	*p < 0.001
Hemoglobin	−0.141	*0.022
Platelet	−0.184	*0.003
Glucose	0.080	0.199
Urea	0.032	0.609
Creatinine	0.018	0.776
Hematocrit	−0.169	*0.006
Procalcitonin	0.393	*p < 0.001
PNI	−0.584	*p < 0.001

All correlation coefficients reported in this table are Spearman’s rank correlation (ρ). All *p*-values are two-sided.

*Denotes statistical significance (*p* < 0.05). PNI, prognostic nutritional index; CONUT, Controlling Nutritional Status; CRP, C-reactive protein; AST, aspartate aminotransferase.

Subsequently, in the logistic regression analysis performed on the development of complications, the model was found to be significant (*p* < 0.001) with a Nagelkerke R^2^ of 0.316, indicating moderate explanatory power. The overall correct classification rate of the model was 72.4% (Table 5). Specifically, amylase (*p* = 0.018), ALP (*p* = 0.016), neutrophils (*p* < 0.001), eosinophils (*p* = 0.030), length of hospitalization (*p* = 0.001), and the presence of other comorbidities (*p* = 0.047) were found to have a statistically significant effect on the development of complications. Accordingly, these parameters were found to affect the development of complications 1.000-fold, 0.994-fold, 1.000-fold, 0.998-fold, 1.146-fold, and 0.556-fold, respectively.

**TABLE 5:** Evaluation of factors affecting complication development by logistic regression.

Parameter	OR	β (SE)	95% CI	*P*
Amylase	1.000	0.0000 (0.0003)	1.000–1.001	*0.018
ALP	0.994	−0.0060 (0.0026)	0.989–0.999	*0.016
Neutrophil	1.000	0.0000 (0.0000)	1.000–1.000	*0.000
Eosinophil	0.998	−0.0020 (0.0013)	0.995–1.000	*0.030
Length of stay	1.146	0.1363 (0.0394)	1.061–1.238	*0.001
Presence of comorbidity	0.556	−0.5870 (0.2953)	0.312–0.993	*0.047
Constant	0.197	−1.6246 (NA)		*0.009

OR, odds ratio; ALP, alkaline phosphatase. NA, not available. Coefficients are unstandardized. ORs are presented per one-unit increase in the original measurement units.

*Indicates statistical significance at *p* < 0.05.

**FIGURE 2:**
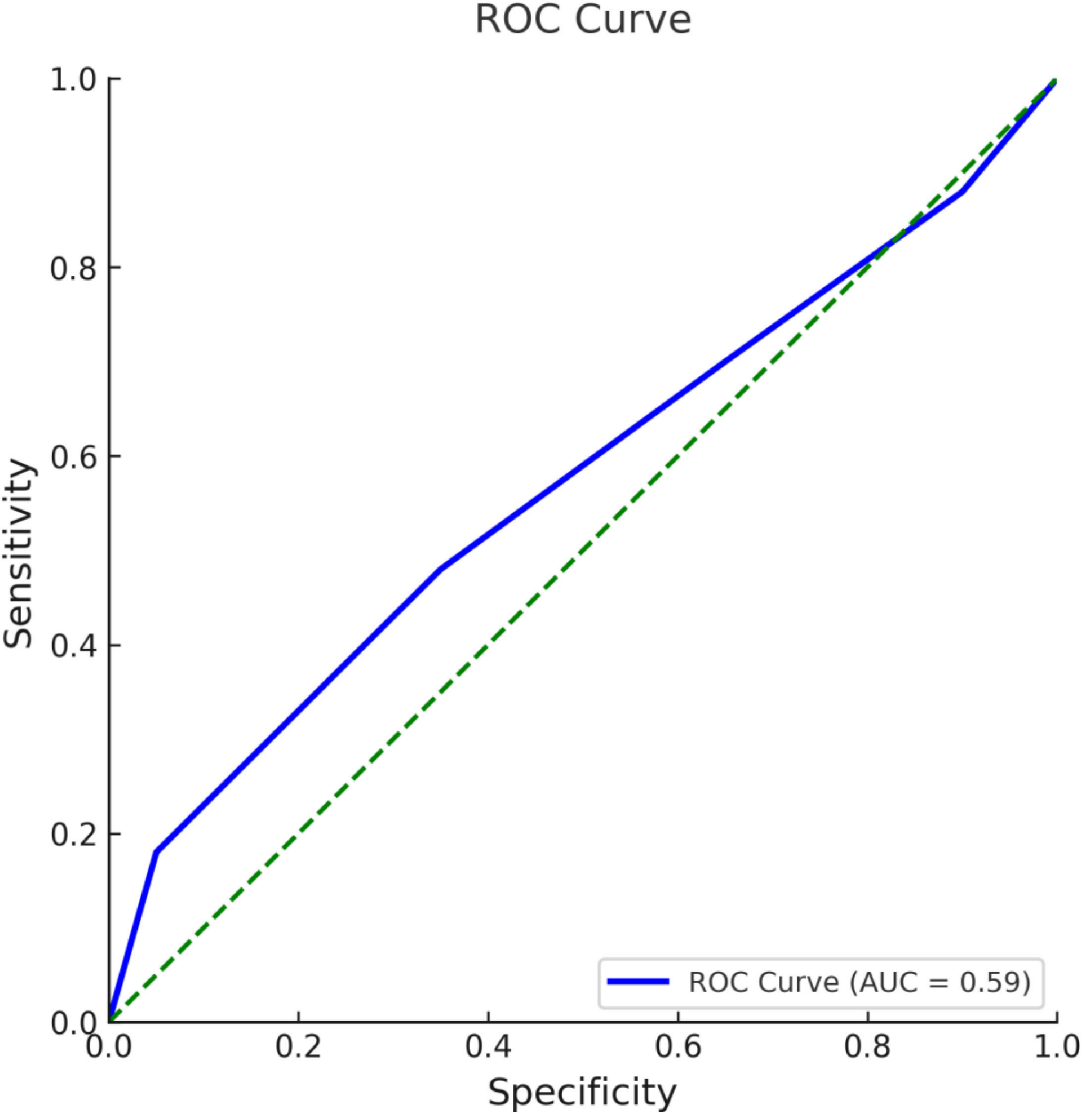
ROC curve for CONUT level in complication development.

In ROC analysis, the area under the curve (AUC) for the CONUT score was 0.588 (95% CI: 0.520–0.657), which was significantly higher than the chance level of 0.5 (*p* = 0.013). The cutoff value for the CONUT level was ≥1.5, and the test’s sensitivity and specificity were calculated to be 67.4% and 47.3%, respectively (Figure 2).

## 4 Discussion

In this study, we investigated the relationship between nutritional status, as assessed by PNI and CONUT scores, and the clinical course of AP. Our findings demonstrated that poor nutritional status, particularly as reflected by higher CONUT scores, was significantly associated with the development of complications, older age, and the presence of necrosis. While PNI alone did not significantly differ between complication groups, its inverse correlation with CONUT and its lower values in necrotic cases suggest its complementary value in assessing nutritional risk. The key findings of our study can be summarized as follows: (i) there was no significant association between PNI score and the development of complications; (ii) higher CONUT scores were significantly associated with complication development; (iii) patients with pancreatic necrosis had significantly lower PNI and higher CONUT scores; (iv) PNI showed a significant inverse correlation with CRP levels, while CONUT did not; and (v) a strong inverse relationship was observed between PNI and CONUT scores. These results suggest that nutritional indices–especially the CONUT score may help identify patients who are more likely to develop complications. The correlation observed between PNI and CONUT is understandable, given that both rely on overlapping laboratory markers, such as albumin and lymphocyte levels. While early enteral nutrition remains the cornerstone of nutritional management in severe AP, the routine use of pharmacometrician is not currently supported due to a lack of evidence for clinical benefit. However, it should be noted that these indices may also be influenced by early intravenous fluid therapy, a standard component of AP management. Hemodilution caused by fluid resuscitation can affect serum albumin and lymphocyte levels, potentially altering PNI and CONUT values in the early phase, regardless of the patient’s actual nutritional status. The CONUT score, as a practical and straightforward tool, may help clinicians detect nutritional risk at an early stage.

Diabetes mellitus (DM) is recognized as a significant risk factor for AP, comparable to other major comorbidities. Several studies have demonstrated that the risk of developing AP is 1.85–2.88 times higher in patients with type 2 diabetes compared to those without (9). In a study by Murata et al. (10), it was found that patients with significant comorbidities experienced a more severe course of pancreatitis and more extended hospital stays. Similarly, Uomo et al. (11) investigated the impact of advanced age and comorbidities on disease progression in 439 patients with AP. Although comorbidities were not directly associated with the development of necrosis, morbidity, or mortality in necrotizing cases, their presence influenced overall disease dynamics. De Campos et al. (12) further supported this by showing a significant relationship between the occurrence of complications and prolonged hospitalization in a cohort of 175 patients treated between 2003 and 2005. In our observations, patients who developed complications often had more extended hospital stays, which aligns with what De and colleagues previously reported. Spotting comorbidities early–right when patients are admitted–might give clinicians a better chance to catch problems early and respond in a way that fits the individual case.

In our study, no significant correlation was found between CRP levels at admission and the development of complications. However, literature reports suggest that CRP, particularly when measured at 48 h (>150 mg/L), has high sensitivity and specificity for predicting severe pancreatitis (13). The absence of 48-h CRP data in our study may explain this discrepancy. We observed an inverse relationship between CRP and PNI scores, suggesting that PNI may reflect both nutritional status and systemic inflammation. This finding is consistent with those of Itami and Fu, who also reported a similar association in patients with a poor prognosis (14, 15). In contrast, no significant correlation was found between CRP and CONUT score. Additionally, elevated glucose levels were significantly correlated with the development of complications, which supports findings from other studies emphasizing the prognostic value of glucose levels in AP (16). However, no significant relationship was found between triglycerides, total cholesterol, or LDL levels and complications, likely due to the exclusion of hypertriglyceridemic patients in our study, which may have hindered the demonstration of lipid profile complications. Elevated glucose levels, which align with complication development, further support its prognostic value in AP. The exclusion of hypertriglyceridemic patients may explain the lack of significant findings regarding lipid profiles.

The PNI, initially defined by Onodera et al. (17) in 1984, was developed to assess nutritional status and surgical risk in patients with gastrointestinal malignancies. In their study involving 200 patients, a PNI score > 45 was considered safe for gastrointestinal surgery, 40–45 indicated moderate risk, and a score < 40 was deemed a contraindication for surgery. Lee et al. (18) later demonstrated in a cohort of 499 patients with pancreatic cancer that a PNI ≤ 46.5 was significantly associated with reduced survival. However, not all studies have shown consistent prognostic value of PNI. For example, Dogan et al. (19) found no significant association between PNI and disease prognosis in 146 patients with metastatic pancreatic cancer. Similarly, Sekine et al. (20) evaluated postoperative outcomes in 116 patients undergoing pancreaticoduodenectomy and reported a significant relationship between CONUT score and prognosis but no such relationship for PNI. In our study, consistent with these findings, the PNI score was not significantly associated with the development of complications in AP. Similar findings were reported by Efgan et al. (21), who observed lower PNI scores in patients with necrotizing pancreatitis; however, PNI alone was not found to be a strong predictor based on ROC analysis. Our results support the idea that while PNI may provide a general sense of nutritional status, it may have limited value in predicting outcomes in acute cases, such as pancreatitis.

Pancreatic necrosis can occur without leading to organ failure in AP, but it still has clinical significance, especially when infection is present. Infected necrosis, in particular, has been linked to a much higher risk of death (3, 22). Despite this, studies investigating the association between local complications–especially necrosis–and nutritional indices such as the PNI score in patients with AP remain limited. In our study, patients with necrosis had significantly lower PNI scores, suggesting a potential link between impaired nutritional and inflammatory status and the development of necrotic complications. Similar trends have been noted in other conditions involving inflammation and cancer, which shows that the link between nutrition and disease severity may extend beyond AP. PNI, in this context, might serve as a marker reflecting both nutritional and inflammatory status.

In literature, several studies have examined the relationship between the PNI and hematological inflammatory markers, particularly the neutrophil-to-lymphocyte ratio (NLR). Shimizu et al. (23) retrospectively analyzed 334 patients with non-small cell lung cancer who underwent surgery and found a significant inverse correlation between preoperative PNI and NLR, suggesting that a lower nutritional status was associated with higher systemic inflammation. Similarly, Xia et al. (24) evaluated 154 patients with stage T1–T2 rectal cancer and investigated the prognostic value of preoperative NLR, platelet-to-lymphocyte ratio (PLR), lymphocyte-to-monocyte ratio (LMR), and PNI. Their findings showed that elevated PLR and LMR, along with low PNI, were significantly associated with increased postoperative morbidity and mortality. In the current study, we observed a significant inverse correlation between PNI and neutrophil count, as well as a positive correlation with lymphocyte, monocyte, and platelet counts. Unlike earlier studies that examined composite ratios, we evaluated the relationship between PNI and individual blood cell counts. We observed that PNI tended to vary about specific immune markers, regardless of other factors. This could suggest that PNI provides insight into a patient’s nutritional and inflammatory state.

In a study conducted by Bakshi et al. (25), which investigated morbidity and mortality following liver transplantation, a significant inverse relationship was identified between the PNI score and both ALT and bilirubin levels. Our results were in line with this, showing similar inverse correlations with ALT and bilirubin). These results suggest that a lower PNI score, indicative of poorer nutritional and inflammatory status, may be associated with greater hepatic dysfunction. These findings indicate that PNI may be influenced not only by nutritional status but also by the extent of hepatic involvement in AP.

One of the prognostic indicators during AP is a decrease in serum calcium level below 8 mg/dL within the first 48 h. For this reason, calcium is commonly monitored in hospitalized AP patients (26). In a retrospective study by Jin et al. (27), 100 pregnant patients diagnosed with AP had their biochemical parameters and PNI scores evaluated about disease severity. The current study found that low PNI scores were correlated with low serum calcium levels. Although no significant difference was observed between the two groups in our study, a significant positive correlation was identified between PNI score and serum calcium level. This supports previous observations that calcium levels may reflect both metabolic disturbance and nutritional condition. This correlation suggests that PNI may reflect not only nutritional status but also specific biochemical and inflammatory changes that are relevant to disease progression in AP.

Most studies in the literature have demonstrated a significant inverse relationship between PNI score and age, indicating that nutritional and immunological status tends to decline with advancing age (14). Our results showed that PNI values were lower in older patients, which is consistent with previous studies reporting age-related changes in nutritional status. Additionally, our analysis revealed a significant positive correlation between the PNI score and eosinophil and basophil counts, as well as a significant inverse correlation with liver enzymes, including AST, ALP, and GGT. These associations have been less commonly discussed in previous studies and may contribute to a better understanding of the nutritional profile in AP. The positive correlation between eosinophils and basophils may reflect a preserved immune response in patients with better nutritional status. At the same time, the inverse relationship with liver enzymes may suggest that hepatic dysfunction is more prevalent in patients with lower PNI scores. These observations suggest that PNI may be associated with both immune function and liver involvement in AP and could be considered in future research on disease monitoring.

Kuroda et al. (28) investigated the prognostic value of the CONUT score in 416 patients who underwent curative resection for gastric cancer. Based on ROC analysis, patients were stratified into high (CONUT ≥ 4) and low (CONUT ≤ 3) groups. They reported significantly lower survival rates in the high CONUT group, highlighting the link between nutritional status and long-term outcomes. Similarly, Li et al. (29) evaluated the prognostic relevance of the CONUT score in a large cohort of 861 patients with resected breast cancer in China. In this study, a CONUT score of ≤2 was classified as low, and a score of ≥3 was classified as high. The sensitivity and specificity of this threshold were reported as 81.6% and 35.7%, respectively. We noticed that patients with higher CONUT scores tend to live shorter, which points to a possible connection between nutrition and cancer outcomes. CONUT might also help assess nutritional risk in other cases, such as AP. In present study, the ROC analysis yielded an AUC of 0.588 for the CONUT score, indicating poor discriminatory ability for predicting complications in acute pancreatitis. Although statistically significant, this modest performance suggests that the CONUT score alone may not be sufficient as a standalone prognostic marker. Combining nutritional indices with other clinical or laboratory parameters could enhance predictive accuracy in future research.

The studies by Akkuzu et al. (30) and Efgan et al. (21) share common ground with our research in that they investigate the relationship between the CONUT and PNI scores and the prognosis of AP. Although our study shares common ground with theirs, the aims and methods were not entirely the same. First, Efgan et al. (21) specifically focused on predicting necrotizing pancreatitis, a severe complication of AP, whereas our study investigated a broader range of complications. While we also observed a correlation between lower PNI scores and necrosis, our study also included various complications, such as pseudocysts and abscesses. Akkuzu et al. examined patients with AP in general terms, whereas we aimed to understand how nutritional scores relate to specific complications and their associated laboratory features. Although all three studies were retrospective, we included nutritional scores alongside laboratory and inflammation data to examine various aspects of the disease.

## 5 Limitations

Although this study covers a 5-years period with broad inclusion criteria, the total number of patients included was relatively low. This may limit the generalizability of the findings. To address this, a *post hoc* power analysis was performed, which indicated that the sample size was sufficient to detect significant associations for the main outcomes. Nonetheless, larger prospective studies are warranted to confirm these results.

Our findings suggest that CONUT and PNI scores could help evaluate prognosis in patients with AP. While PNI was not significantly correlated with the development of complications, CONUT scores were considerably higher in patients with complications, particularly those involving necrosis, suggesting that nutritional status may influence disease progression. PNI was often lower when CRP levels were higher, which could indicate that individuals with inflammation also have poor nutrition. This makes it more important to consider nutrition early, and CONUT might help identify those who could have more significant problems. These findings address a gap in literature by highlighting the prognostic value of combined nutritional indices in AP. Future studies should focus on larger, prospective cohorts and investigate the impact of targeted nutritional interventions guided by these scores to improve patient outcomes.

## Data Availability

The original contributions presented in this study are included in this article/supplementary material, further inquiries can be directed to the corresponding author.
